# Circadian Rhythms and Breast Cancer: The Role of Per2 in Doxorubicin-Induced Cell Death

**DOI:** 10.1155/2015/392360

**Published:** 2015-08-11

**Authors:** Megan I. Mitchell, Anna-Mart Engelbrecht

**Affiliations:** Department of Physiological Sciences, Stellenbosch University, Stellenbosch, Matieland 7602, South Africa

## Abstract

Mammalian circadian rhythms form an integral physiological system allowing for the synchronisation of all metabolic processes to daily light/dark cycles, thereby optimising their efficacy. Circadian disruptions have been implicated in the onset and progression of various cancers, including those arising in the breast. Several links between the circadian protein Per2 and DNA damage responses exist. Aberrant Per2 expression results in potent downstream effects on both cell cycle and apoptotic targets, suggestive of a tumour suppressive role for Per2. Due to the severe dose limiting side effects associated with current chemotherapeutic strategies, including the use of doxorubicin, a need for more effective adjuvant therapies to increase cancer cell susceptibility has arisen. This study was therefore aimed at characterizing the role of Per2 in normal breast epithelia (MCF-12A) and in ER^−^ breast cancer cells (MDA-MB-231) and also at determining the role of Per2 in doxorubicin-induced cell death. In both cell lines Per2 protein expression displayed a 24-hour circadian rhythm in both cell lines. Per2 was located predominantly in the cytoplasm, with nuclear localization observed with lower cytoplasmic fluorescent intensities. Our results show that Per2 silencing effectively sensitizes the chemoresistant MDA-MB-231 breast cancer cells to the cytotoxic effects of doxorubicin.

## 1. Introduction

The carcinogenic process is fundamentally complex and highly variable, with no single genetic alteration giving rise to cancer. However, the initiation of cancer development encompasses a series of stages beginning with an initial driver mutation responsible for tumourigenesis, followed by an accumulation of additional genetic mutations, conferring both proliferative and survival advantages [1]. Cancer cells have thus developed a variety of exquisite mechanisms to evade cell death [2]. As such, current anticancer strategies involve the use of either radiation or chemotherapeutic agents, like doxorubicin (Dox), for the treatment of various solid tumours. However, aside from the severe cumulative dose-dependent side effects associated with the use of anthracycline antibiotics like Dox, resistance of cancer cells to chemotherapeutic strategies (chemoresistance) has become an ongoing complex issue faced by many cancer patients. Currently it is believed that chemoresistance accounts for over 90% of the failure rate seen with the treatment of metastatic breast cancer (MBC) [3]. Thus, a critical need for new treatment approaches, which increase the susceptibility of these resistant cancer cells to chemotherapeutic strategies, has arisen.

According to the World Health Organization's (WHO) International Agency for Research on Cancer (IARC) a wide range of human epidemiologic evidence suggests that circadian disruption brought on by shift work is most likely carcinogenic to humans (IARC classification, Group 2A) [4]. Furthermore, evidence also suggests that the synchronization of circadian rhythms may influence antitumour tolerability and the pharmacological efficacy of chemotherapeutic drugs [5].

Circadian rhythms are external manifestations of intrinsic biological time measuring cycles on a 24-hour scale [6]. To date, all mammalian cell types have been shown to possess an intrinsic circadian clock, made up of self-sustained and self-perpetuating transcriptional feedback loops, responsible for keeping time within the cell [7]. Although the internal circadian rhythms of mammals have been known for centuries [8], the molecular nature behind these oscillations has only recently been understood. Central to the correct functioning of the circadian rhythm are the basic helix-loop-helix PER-ARNT-SIM (PAS) domain proteins Bmal1 and CLOCK, which heterodimerize [9], and ultimately lead to the expression of their repressors: period (Per1, Per2, and Per3) and cryptochrome (Cry1 and Cry2) [10]. Upon translation Per and Cry proteins heterodimerize and associate with human casein kinase 1 *ε* (CK1*ε*). This complex translocates into the nucleus resulting in the inhibition of CLOCK:Bmal1 mediated transcription [11].

A study conducted by Filipski and colleagues demonstrated that a severe disruption in the central circadian clock of tumour-bearing mice resulted in accelerated tumour growth, confirming known clinical results where patients with maintained 24-hour circadian rhythms generally have an improved prognosis compared to those with disrupted circadian rhythms [12]. Additionally, a wide variety of clinical and* in vivo *evidences suggest that the circadian timing of when chemotherapeutic agents are administered plays a critical role in the pharmacological efficacy and toxicity of both doxorubicin and cisplatin [13]. It is, therefore, evident that further investigations into the effects of circadian clock disruption on the cellular DNA damage response and cancer susceptibility are warranted, specifically with regard to elucidating the mechanisms behind this circadian clock disruption.

Additionally, cell cycle genes have recently been shown to be regulated directly by circadian clocks [14]. However, circadian clocks operate accurately independent of cell cycle regulation [15]. Currently little is known about the exact mechanisms of how circadian clocks regulate cell cycle progression. Therefore this study was designed to provide insight into the molecular nature behind intrinsic peripheral circadian clocks, as well as assess whether the manipulation of these clocks through the silencing of Per2, a key regulator involved in the resetting of the circadian clock, could sensitize resistant breast cancer cells to the anticarcinogenic effects of Dox.

## 2. Materials and Methods

### 2.1. Cell Culture

MDA-MB-231 breast cancer cells were cultured in Dulbecco's Modified Eagles Medium (DMEM) supplemented with 10% Foetal Calf Serum (FCS) and 1% Penicillin Streptomycin (standard growth medium). The noncancerous MCF-12A cells were cultured in a 1 : 1 ratio of DMEM and Ham's F-12 nutrient mixture supplemented with 10% Foetal Calf Serum and 1% Penicillin Streptomycin, 10 *μ*g/mL Insulin (Humulin 30/70), 20 ng/mL Epidermal Growth Factor (EGF), 500 ng/mL hydrocortisone, and 100 ng/mL cholera toxin. Cells were maintained at an atmosphere of 37°C and a humidity 5% CO_2_ and regularly subcultured once a confluency of 70–80% was reached. Upon reaching 80% confluency, cells were split and seeded with fresh media for experiments.

### 2.2. Treatments

Prior to treatment, media were aspirated and cell monolayer was washed twice with warm PBS to ensure all cell debris was removed. The concentration of doxorubicin (2.5 *μ*M) employed was selected based on cell viability, with the highest concentration that produces the least toxic effect in normal (MCF-12A) cells while still increasing cell death in the cancer (MDA-MB-231) cells used.

### 2.3. esiRNA Knockdown of Per2

Cells were plated into 6-well plates in standard medium 24 hours prior to transfection. Endoribonuclease-prepared siRNA (esiRNA) targeted against Per2 (Sigma, EHU070571) was freshly prepared in serum-free media containing the transfection reagent (HiPerfect). The mixture was allowed to incubate at room temperature for 20 minutes in order for esiRNA:HiPerfect complexes to form. Antibiotic-free growth media were then added to produce a final concentration of 30 nM, which was added dropwise onto cells. Cells were incubated at an atmosphere of 37°C and 5% CO_2_ humidity for 48 hours before subsequent treatment with 2.5 *μ*M Dox.

### 2.4. SDS-PAGE and Western Blot Analysis

Cells were washed twice with cold PBS and lysed on ice using modified radioimmunoprecipitation assay (RIPA) buffer (2.5 mM Tris-HCL, 1 mM EDTA, 1 mM dithiothreitol, 0.1 mM phenylmethylsulfonyl fluoride (PMSF), 1 mM benzamidine, 50 mM NaF, 4 mg/mL SBTI, 10 mg/mL leupeptin, 0.1% SDS 0.5%, Na deoxycholate, and 1% NP40, calibrated to pH 7.4). Whole cell lysates were sonicated on ice at 3 Hz for short bursts and centrifuged at 4°C for 10 min at 8000 RPM. Cell lysates were subsequently separated on 4–15% polyacrylamide precast gels (mini-PROTEAN TGX Gels, Bio-Rad) by sodium dodecyl sulphate polyacrylamide gel electrophoresis (SDS-PAGE) at 100 V for ±90 minutes. On completion of SDS-PAGE, proteins were transferred to prepared polyvinylidene fluoride (PVDF) membranes using a semidry electrotransfer system (TransBlot Turbo v1.02, BioRad) for 30 minutes at 25 V. Membranes were stained with Ponceau S to visualize protein transfer and nonspecific binding was prevented by blocking membranes in 5% (w/v) fat-free milk in Tris-buffered saline- (TBS-) T (1x TBS, pH 6.8, 0.1% Tween-20) for 1 hour. Membranes were incubated at 4°C overnight with TBS-T diluted anti-rabbit primary antibodies (1 : 1000) targeted against Per2 (Abcam, ab64460), cleaved caspase-3 (Cell Signalling, #9661), and cleaved PARP (Cell Signalling, #5625). Membranes were washed with TBS-T (3 × 5 min) before being incubated in anti-rabbit IgG horseradish peroxidase conjugated secondary antibody (1 : 10000) with gentle agitation at room temperature for 1 hour. Membranes were washed before proteins were detected with the use of an ECL western blotting substrate detection kit (Pierce, Thermo Scientific) and protein bands visualised with the use of ImageLab 4.0 software on a Chemi-Doc MP (BioRad) imaging system. Exposed bands were visualized and quantification was done using densitometry with the use of Quantity One densitometry software. Bands for each specific protein were quantified as density readings comparative to the control sample.

### 2.5. Immunocytochemistry

MCF-12A and MDA-MB-231 cells were cultured in 8-well chamber slides (Thermoscientific) at a density of 12 000 cells/well. After the treatment period, growth medium was removed and cells were washed twice with warm PBS. Cells were fixed with ice cold methanol and acetone (1 : 1). The fixative mixture was then removed and cells were rinsed with Phosphate Buffered Saline (PBS); nonspecific binding was prevented by incubating cells in a 1% BSA/PBS blocking solution. After incubation, the blocking solution was drained off and a primary antibody against Per2 (Abcam, ab64460) diluted in PBS (1 : 50) was added to cells and allowed to incubate overnight at 4°C. Cells were then rinsed with PBS and allowed to incubate with an Alexa Fluor 488 conjugated secondary antibody (Life Technologies). Hoechst 33342 dye (1 : 200) was additionally added and kept in contact with cells for a further 10 min. Next, cells were rinsed and mounted with DAKO fluorescent mounting medium and analysed on a Zeiss confocal microscope.

### 2.6. MTT Assay

A MTT cell viability assay was conducted to assess the percentage of metabolically viable cells. Subsequent to esiRNA or Dox treatment cells were washed with warmed Phosphate Buffered Saline (PBS) and incubated in MTT (0.001 g MTT/mL PBS) for 90 min at an atmosphere of 37°C, 5% CO_2_ humidity. MTT formazan crystals were dissolved using an Isopropanol:HCl/Triton-X-100 solution. Plates were then analysed using KCjunior software on a universal microplate reader (EL800, Bio-Tek Instruments Inc.) where absorbance values were determined at a wavelength of 540 nm. Absorbance values were all expressed as a percentage of MTT reduction versus the untreated control (100%).

### 2.7. Cell Cycle Analysis

Cell cycle analysis was performed by flow cytometry using the CycleTEST PLUS DNA reagent kit (Becton Dickinson, California, USA). Subsequent to treatment MDA-MB-231 cells were washed, trypsinized, and centrifuged at room temperature for 5 min at 300 ×g. Cells were then counted using a haemocytometer and concentrations were adjusted to 1 × 10^6^ cells/mL using the provided buffer solution. In order to stain prepared cells, cell suspensions were centrifuged at 400 ×g for 5 min at room temperature. Each sample was incubated in a trypsin buffer followed by incubations in a trypsin inhibitor and RNase buffer and finally in an ice cold propidium iodide (PI) stain solution. Samples were analysed on the flow cytometer (BD FACSAria I) within 3 hours of adding the PI staining solution. At minimum of 30 000 list-mode data events were acquired for each sample. ModFit LT software (Verity Software House, Inc., ME, USA) was used to determine the percentage of cells in the G0/G1, S, and G2/M phases. The percentage of apoptotic cells was also determined using ModFit LT software.

### 2.8. G2/M Analysis

After all treatments MDA-MB-231 cells were detached from flasks with 0.25% trypsin EDTA (Life Technologies). The cell suspension was then centrifuged at 1500 RPM for 3 min. The supernatant was removed and the pellet resuspended in 1 mL PBS. Formaldehyde at a final concentration of 4% was added and cells were fixed at 37°C for 10 min, after which cells were placed on ice. Fixative was removed and cells were centrifuged and the supernatant was removed as previously. Permeabilization was achieved by adding ice cold methanol (90%) and incubating cells on ice. Incubation buffer (0.5 g BSA dissolved in 100 mL PBS) was added to cells and rinsed twice by centrifugation. Phospho-Aurora A (Thr^288^)/Aurora B (Thr^232^)/Aurora C (Thr^198^), Alexa Fluor 488 conjugated (Cell Signalling Technologies) and Phospho-Histone H3 (Ser^10^), and Alexa Fluor 647 conjugated (Cell Signalling Technologies) primary antibodies were added to cells (1 : 50 dilution in incubation buffer) and incubated for 1 hour at room temperature. Cells were washed by centrifugation in incubation buffer before being resuspended in 0.5 mL PBS and analysed on the flow cytometer (BD FACSAria I). A minimum of 30 000 events was collected and analysed using a 488 nm laser and 502LP, 520/30BP emission filters.

### 2.9. Statistical Analysis

All statistical analysis was carried out using GraphPad Prism 5. All data was assessed using mean ± SEM. One way analysis of variance (ANOVA) with Bonferroni post hoc corrections as well as Mann-Whitney *U* *t*-tests was conducted where appropriate. A *P* value < 0.05 was considered statistically significant.

## 3. Results

### 3.1. Characterizing the Role of Per2 in Normal Breast Epithelia as well as in ER^−^ Cancer Cells

**Figure 1:**
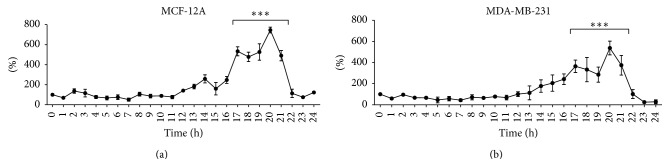
Rhythmic expression of the mammalian circadian protein period 2 (Per2) in nontumourigenic breast epithelial MCF-12A cells (a) and MDA-MB-231 estrogen receptor negative human breast adenocarcinoma cells (b). MCF-12A and MDA-MB-231 cells were harvested hourly for a period of 25 hours (Time 0 = baseline). Western blot analysis was employed to assess relative Per2 protein levels. Values (normalized to GAPDH) are expressed as a percentage of baseline and presented as mean ± SEM (*n* = 3). *∗∗∗* = *P* < 0.0001 versus baseline.

**Figure 2:**
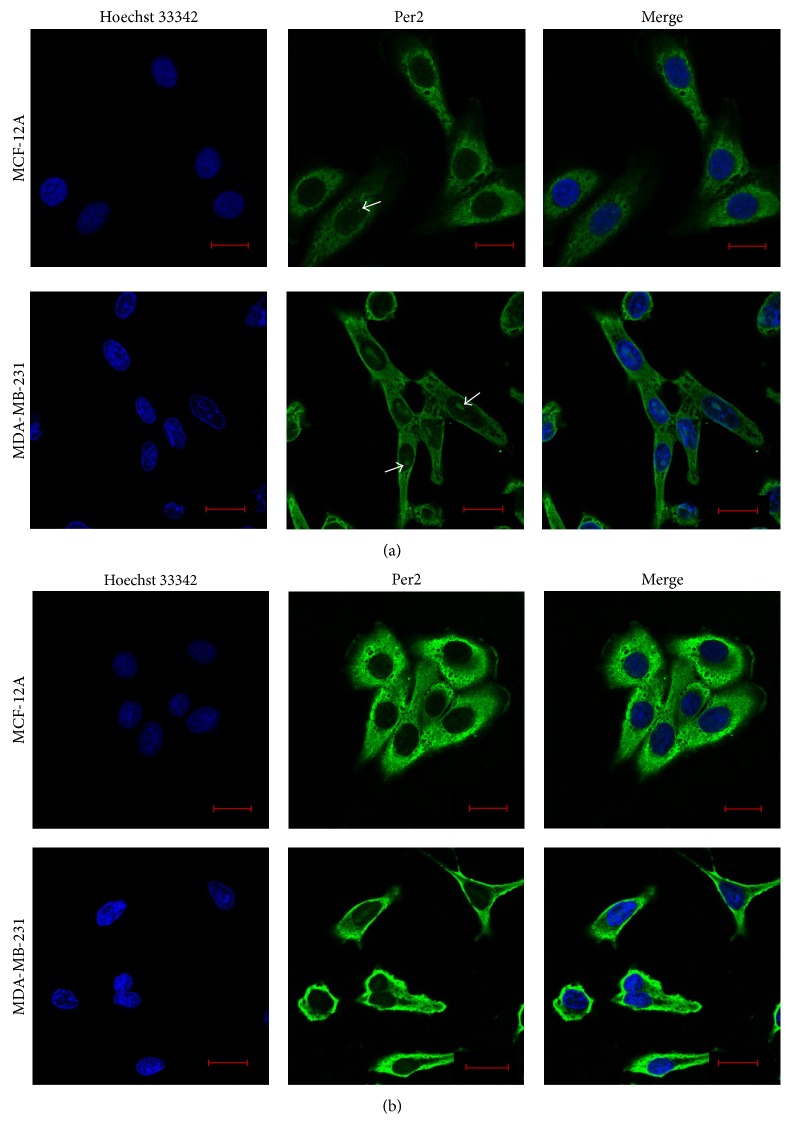
Determination of Per2 localization. Both MCF-12A and MDA-MB-231 cells were cultured under standard control cellular conditions in 8-well chamber plates for a period of 24 hours. Cells were immunostained for Per2 (Green) and Hoechst 33342 (Blue) and visualized using fluorescent confocal microscopy at a 63x magnification. Two distinct populations of Per2 localization were observed in both the MCF-12A and MDA-MB-231 cell lines: (a) Per2 nuclear localization was seen in the fluorescently dimmer cell populations; MCF-12A cells show slight nuclear localization; however this was more prominent within the MDA-MB-231 breast cancer cells. White arrows indicate nuclear localization of Per2. (b) Per2 was localized exclusively within the cytoplasm of the fluorescently brighter cell population in both cell lines.

To determine the presence of a circadian expression pattern in the protein levels of Per2 we characterized the relative protein concentration of Per2 over time. Cells were cultured under standard cell culture conditions and protein extractions were carried out hourly for a period of 25 hours commencing at 07h00 and terminating the following day at 08h00. MCF-12A cells show a clear circadian pattern in Per2 protein expression with a significant increase in Per2 protein levels seen at 20 hours (03h00) when compared to baseline (0 hours = 07h00). The MDA-MB-231 cells showed the same rhythmic expression pattern for Per2, with levels significantly increasing at 20 hours (03h00) when compared to baseline (0 hours = 07h00), however, to a much lesser extent than that observed in the MCF-12A cells (Figure 1). To characterize the cellular localization of Per2, both MCF-12A and MDA-MB-231 cells were immunostained with Per2:Alexa Fluor 488 and Hoechst 33342 and visualized by means of confocal fluorescent microscopy. The MCF-12A breast epithelial cells show a predominant localization of Per2 within the cytoplasm, with slight colocalization in the nucleus, whereas the MDA-MB-231 cancer cells show a more prominent nuclear colocalization of Per2 (Figure 2). Additionally both cell lines display two distinct populations of Per2 fluorescent intensities, a dim population (Figure 2(a)) as well as a brighter more fluorescently intense population (Figure 2(b)). In both cell lines it was noted that Per2 nuclear localization was limited to the fluorescently dimmer subpopulation of cells.

### 3.2. The Role of Per2 in Doxorubicin-Induced Cell Death in the MDA-MB-231 Cancer Cells

**Figure 3:**
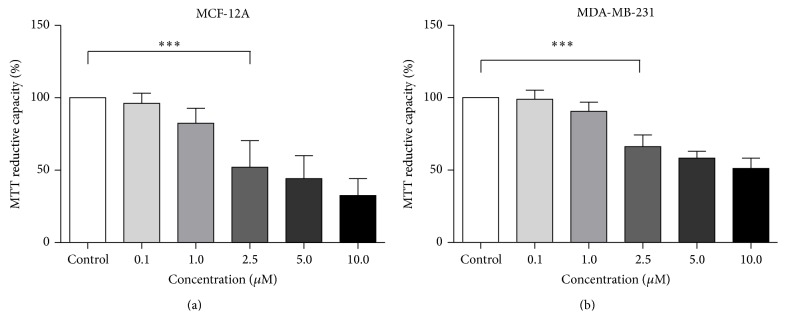
The effect of various concentrations of doxorubicin on the viability of nontumourigenic breast epithelial MCF-12A cells (a) and triple negative breast cancer cells MDA-MB-231 (b). MCF-12A and MDA-MB-231 cells were incubated in 0 (control), 0.1, 2.5, 5.0, and 10.0 *μ*M doxorubicin for 24 hours. Cell viability was assessed using the MTT assay. Values are expressed as a percentage of the control and presented as mean ± SEM (*n* = 3). *∗∗∗* = *P* < 0.0001 versus control.

Clinically the administration of doxorubicin involves the intravenous bolus infusion of doses ranging between 15 and 90 mg/m^2^, which results in plasma concentrations ranging from 0.3 to 5 *μ*M [16]. Our results demonstrated that 24-hour treatment with 5 *μ*M of doxorubicin resulted in a significant reduction in the cell viability of the chemoresistant MDA-MB-231 cancer cells (Figure 3), but as this concentration falls at the extreme limit of the clinically relevant dose range, we aimed to use a lower dose which still falls within this clinically relevant range. Based on these results as well as the fact that 2.5 *μ*M falls within the range of clinically relevant dosages for doxorubicin, for this study we used 2.5 *μ*M for all subsequent doxorubicin treatments.

**Figure 4:**
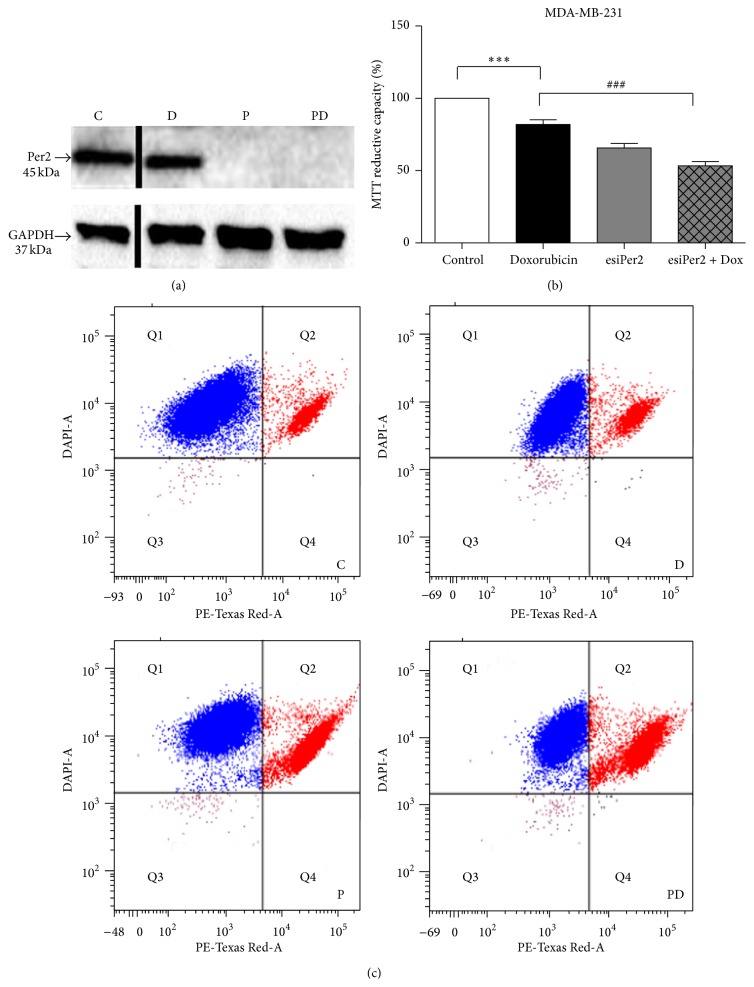
Determining the effects of Per2 silencing on doxorubicin-induced cell death in the ER^−^ MDA-MB-231 breast cancer cells. MDA-MB-231 cells were subjected to (1) control conditions (C), (2) 2.5 *μ*M doxorubicin (D), (3) 30 nM Per2 esiRNA (P), and (4) 30 nM Per2 for 48 hours + 2.5 *μ*M doxorubicin for 24 hours (PD). (a) Representative western blots of Per2 protein expression in the MDA-MB-231 cancer cells following treatments. (b) Cell viability was assessed using the MTT assay. Values are expressed as a percentage of the control and presented as mean ± SEM (*n* = 3). *∗∗∗* = *P* < 0.0001 versus control and ### = *P* < 0.0001 versus doxorubicin. (c) Representative flow cytometry box plots of the MDA-MB-231 breast cancer cells obtained by Hoechst 33342 and propidium iodide (PI) staining following treatments. Q1 represents cells staining positive for Hoechst and negative for PI (live cells) and Q2 represents cells staining positive for both Hoechst and PI (dead cells).

Per2 protein expression was silenced in combination with Dox in order to determine whether the chemoresistant MDA-MB-231 cancer cells can be sensitized to Dox treatment. A 98.5% silencing efficiency was achieved in the MDA-MB-231 cancer cells following 30 nM esiRNA treatment for 48 hours (Figure 4(a)). MTT assays revealed a significant reduction in mitochondrial reductive capacity following treatment with Dox alone, which was further enhanced with Per2 silencing prior to Dox treatment (Figure 4(b)). These results were further supported by means of flow cytometry where Per2 silencing prior to Dox treatment significantly increased the susceptibility of the MDA-MB-231 cancer cells to Dox-induced cell death (Figure 4(c)).

**Figure 5:**
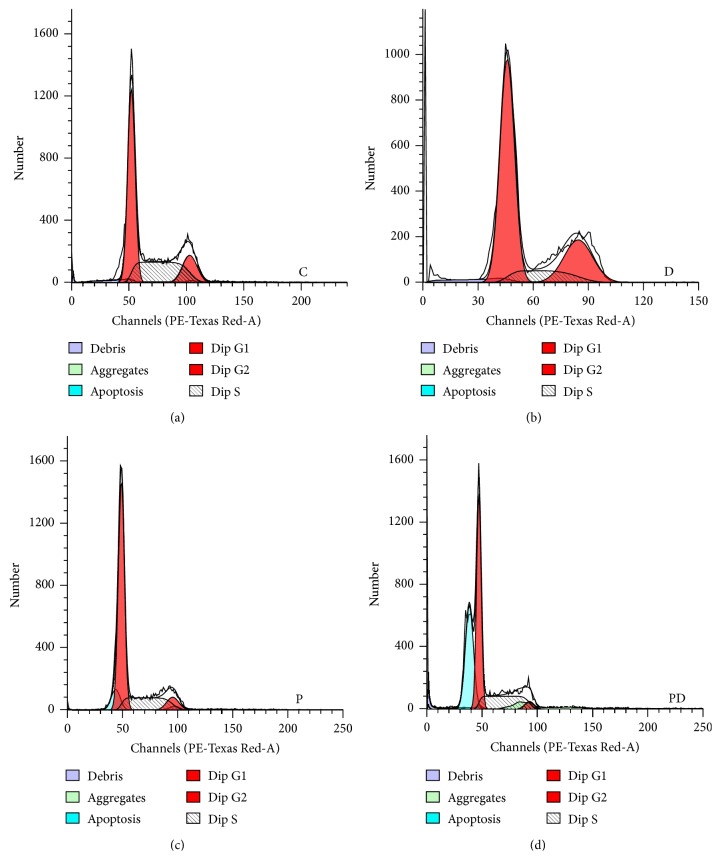
Analysis of cell cycle progression in MDA-MB-231 breast cancer cells. MDA-MB-231 cells were subjected to (1) control conditions (C), (2) 2.5 *μ*M doxorubicin (D), (3) 30 nM Per2 esiRNA (P), and (4) 30 nM Per2 for 48 hours + 2.5 *μ*M Dox for 24 hours (PD). Cell cycle analysis was assessed following propidium iodide staining by flow cytometry. Statistical analysis: one way ANOVA with Bonferroni post hoc correction. All results are presented as mean ± SEM (*n* = 3). *P* < 0.05 versus control and *P* < 0.001 versus control.

**Figure 6:**
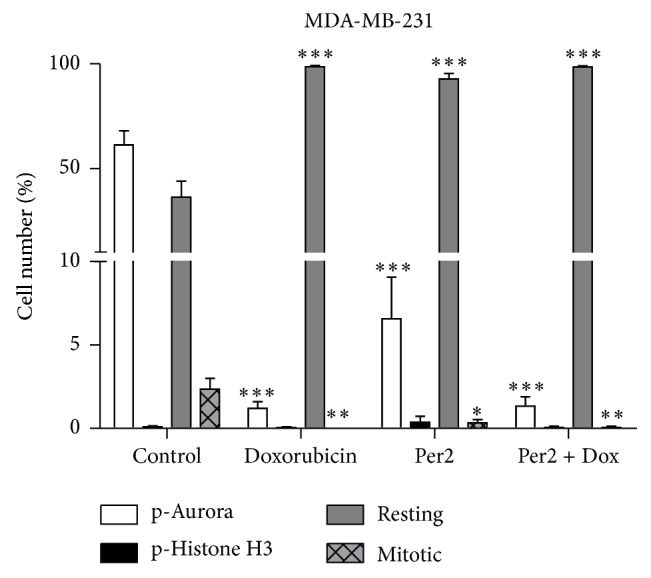
Determination of G2/M cell cycle transition in MDA-MB-231 breast cancer cells following Per2 silencing and doxorubicin treatment. MDA-MB-231 cells were subjected to (1) control conditions (C), (2) 2.5 *μ*M doxorubicin (D), (3) 30 nM Per2 esiRNA (P), and (4) 30 nM Per2 for 48 hours + 2.5 *μ*M doxorubicin for 24 hours (PD). G2/M transition analysis was assessed by flow cytometry. Statistical analysis: one way ANOVA with Bonferroni post hoc correction. All results are presented as mean ± SEM (*n* = 3). *∗* = *P* < 0.05 versus control, *∗∗* = *P* < 0.001 versus control, and *∗∗∗* = *P* < 0.0001 versus control.

As cell cycle regulation is thought to be under the control of circadian genes, cell cycle analysis using flow cytometry was assessed to determine the effects of Per2 silencing on the ability of cells to progress through the cell cycle. Our results revealed that Per2 silencing alone leads to G0/G1 cell cycle arrest whereas Per2 silencing in combination with Dox leads to arrest in the S-phase of the cell cycle with a significant increase in apoptosis (Figure 5). Furthermore, G2/M transition analysis using conjugated primary antibodies against phosphorylated Histone H3 (Ser^10^) and phosphorylated Aurora A (Thr^288^)/Aurora B (Thr^232^)/Aurora C (Thr^198^) was assessed, in order to distinguish between cells in interphase, early mitosis, and late mitosis. Our results revealed a shift from early mitosis as well as a complete blunting of end stage mitosis with Dox treatment alone as well as in combination with Per2 silencing (Figure 6).

**Figure 7:**
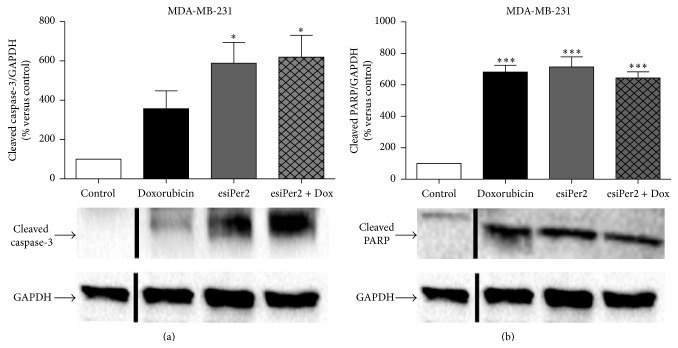
Determining the role of Per2 in caspase-dependent apoptosis. MDA-MB-231 cells were subjected to (1) control conditions (C), (2) 2.5 *μ*M doxorubicin (D), (3) 30 nM Per2 esiRNA (P), and (4) 30 nM Per2 for 48 hours + 2.5 *μ*M Dox for 24 hours (PD). Whole cell lysates were subjected to immunoblotting with appropriate antibodies for cleaved caspase-3 (a) and PARP (b). Statistical analysis: one way ANOVA with Bonferroni post hoc correction. All results are presented as mean ± SEM (*n* = 3). *∗* = *P* < 0.05 versus control and *∗∗∗* = *P* < 0.0001 versus control.

To evaluate the mechanism whereby Per2 silencing increases cell death in the MDA-MB-231 cancer cells, we evaluated the cleavage of the apoptotic markers caspase-3 and poly-ADP-ribose polymerase (PARP) with the use of western blotting. Once caspase-3 is cleaved and activated by upstream caspases in the caspase cascade, apoptotic cell death ensues. A significant increase in cleaved caspase-3 (Figure 7(a)) and cleaved PARP levels were observed following Per2 silencing alone as well as in combination with Dox treatment. Dox is known to induce cell death via a caspase-3 dependent mechanism thus the increase in caspase-3 cleavage corresponds to the increased cleavage of PARP observed following Dox treatment alone (Figure 7(b)).

## 4. Discussion

All nucleated cells have been shown to possess intrinsic circadian clocks, which are thought to function in a cell-autonomous fashion, evident by the self-sustained rhythmic oscillations in detached cell culture models [17]. Therefore, the first part of this study was aimed at elucidating the presence of rhythmic expression patterns of the circadian Per2 protein in a normal breast epithelial (MCF-12A) cell line and in an estrogen receptor negative (ER^−^) cell line (MDA-MB-231) (Figure 1). To our knowledge we have, for the first time, demonstrated a clear circadian pattern in Per2* protein* expression in the normal MCF-12A cells as well as in the ER- MDA-MB-231 cancer cells exists.

Two distinct subpopulations of cells expressing the Per2 protein were observed in both the normal MCF-12A breast epithelial cells and the MDA-MB-231 breast adenocarcinoma cells. In both the fluorescently dimmer and brighter subpopulations, Per2 was seen to be located predominantly within the cytoplasm (Figure 2). Colocalization of Per2 in the nucleus was only seen in the fluorescently dimmer subpopulations of both cell lines. These results are in agreement with known literature where both cytoplasmic and nuclear localization of Per2 are seen in the kidney fibroblast-like COS7 cell line [18]. Additionally, the differences in Per2 protein expression patterns seen in different populations of the MDA-MB-231 cancer cells highlight the asynchronous nature of peripheral circadian clocks as well as the heterogeneity of cancer cell populations [19]. The fact that nuclear localization of Per2 was associated only with diminished cytoplasmic protein expression may be explained by the fact that Per2 protein levels begin to accumulate within the cytoplasm after its translation at the start of a circadian day [20]. Per2 protein levels reach threshold levels at the end of a circadian day, where they heterodimerize with accumulated Cry proteins and CKI*ε*; this complex then translocates into the nucleus resulting in the repression of Per2 gene transcription. Once repressed, unbound cytoplasmic Per2 is phosphorylated by CKI*ε*, leading to its degradation by the 26S proteasome pathway, thus causing a decrease in cytoplasmic Per2 protein levels and a resetting of the circadian clock [21].

The Per2 gene is an essential regulator of the mammalian circadian clock system, and mutations arising in this gene have been identified in a wide range of human cancers including colorectal and breast cancer [22]. Furthermore, circadian Per2 disruption has been implicated in cell cycle dysfunction and apoptosis, evident by the aberrant rhythmic expression of the cell cycle gene, cyclin D1, as well as the negative p53 regulator, MDM2 [23]. Based on this knowledge, we then focused on Per2 as a molecular target for the sensitization of resistant breast cancer cells to Dox treatment.

We show that a lower, clinically relevant, dose of 2.5 *μ*M Dox alone is less effective in inducing cell death in the MDA-MB-231 cells, apparent by a slight reduction in MTT reductive capacity and loss of membrane integrity, effectively demonstrating the resilient nature of these cancer cells to Dox treatment (Figure 3). Our results also indicate that by silencing Per2 prior to treatment with Dox, the resistant MDA-MB-231 cancer cells become more sensitive to the cytotoxic effects of Dox, as both MTT reductive capacity and membrane integrity were significantly reduced in these cells when compared to Dox treatment alone (Figure 4(b)). These results are further supported by a significant increase in caspase-3 and PARP cleavage following combination treatment (Figure 7). These results are somewhat contradictory to others, which show an increase in cell death with the overexpression of Per2 in MCF-7 breast cancer cells [9]. This may in part be explained by the fact that both apoptotic and cell cycle processes display robust circadian organisation, wherein the expression patterns of proteins such as BCL-2 and BAX are under rhythmic circadian control. BCL-2/BAX rhythmic expression patterns have been shown to ultimately affect the antitumour efficacy and toxicity of chemotherapeutic agents, like docetaxel, largely dependent on the circadian phase of the cell cycle [24]. Furthermore, it is plausible that a compensatory mechanism exists to counteract a decrease in Per2 protein expression. An accumulation of Bmal1 resulting from the loss of Per2s inhibitory effect could account for the increase in cell death we observed, as the overexpression of Bmal1 has been shown to increase the sensitivity of oxaliplatin both* in vitro *and* in vivo* [25].

A significant increase in the G0/G1 phase with a concomitant decrease in the G2/M cell cycle phase was seen with Per2 silencing, whereas the combination of Per2 silencing and Dox treatment resulted in cells exiting the cell cycle during the S-phase; this resulted in a significant reduction in the G2/M phase with a concomitant increase in apoptosis (Figure 5). G2/M cell cycle transition was assessed, in order to shed further light on the cell cycle data obtained, particularly during mitosis. The phosphorylation of Histone H3 at the Ser^10^ residue is strongly associated with the condensation of chromosomes during mitosis [26], whereas the phosphorylation of the Aurora kinases (A, B, and C) is associated with various functions during mitosis, which include but are not limited to mitotic spindle formation, chromosome segregation, and cytokinesis [27]. The functional influences of these kinases thus span from the beginning of the G2 phase right to the end of mitosis. Additionally, during G2/M cell cycle transition the phosphorylation of Histone H3 is tightly coupled to the expression of the Aurora kinases [28]. Therefore, cells staining positive for both p-Histone H3 (Ser^10^) and p-Aurora A (Thr^288^)/Aurora B (Thr^232^)/Aurora C (Thr^198^) indicate cells which are truly mitotic and have progressed through the entire cell cycle (late stage mitosis), whereas those staining positive for only the phosphorylated Aurora kinases are indicative of early mitotic cells. Per2 silencing results in a significant decrease in late stage mitosis as well as that of early mitosis; however the decrease in early mitosis was not as severe as that seen with Dox treatment alone (Figure 6). The combination of Per2 silencing and Dox also resulted in a complete inhibition of both early and late mitosis. Our results support the notion that Per2 may be involved in the regulation of cell cycle progression as suggested in literature, and this mechanism may be mediated by the effect of Per2 on cyclin D1 and* c-myc* expression [12]. However, as we did not assess expression of these key G0/G1 cell cycle regulators in response to Per2 silencing, this still remains to be elucidated.

Collectively, results obtained for the genetic modulation of Per2 demonstrate that the resistant nature of the MDA-MB-231 breast cancer cells to Dox treatment was effectively abrogated via the disruption of the intrinsic circadian clock system. The suppression of the 45 kDa Per2 protein by means of a targeted esiRNA approach, in the context of Dox-induced cell death, resulted in cell cycle arrest of the MDA-MB-231 breast cancer cells, with a concomitant increase in apoptosis. These findings support known clinical data whereby the pharmacological efficacy and toxicity of Dox are profoundly coordinated by intrinsic circadian rhythms [29], demonstrating their clinically relevant implications.

Furthermore, as both aberrant Per2 expression [30] and Dox [31] have been shown to induce cellular senescence in response to genotoxic stress, we propose that the induction of apoptosis seen following the sensitization of MDA-MB-231 breast cancer cells to the cytotoxic effects of Dox may in part be mediated by mitotic catastrophe following cellular senescence. However, the underlying mechanisms behind senescence induced cell death in resistant breast cancer cells warrant further investigation.

In conclusion, our results demonstrate the influential role Per2 plays in the circadian efficacy and toxicity of the most important chemotherapeutic agent used in the treatment of cancer to date. The unexplored and highly novel avenue of the circadian clock genes, including Per2, as adjuvant cancer targets warrants further investigation.

## References

[1] Fernald K., Kurokawa M. (2013). Evading apoptosis in cancer. *Trends in Cell Biology*.

[2] Hanahan D., Weinberg R. A. (2000). The hallmarks of cancer. *Cell*.

[3] Coley H. M. (2008). Mechanisms and strategies to overcome chemotherapy resistance in metastatic breast cancer. *Cancer Treatment Reviews*.

[4] Straif K., Baan R., Grosse Y. (2007). Carcinogenicity of shift-work, painting, and fire-fighting. *The Lancet Oncology*.

[5] Filipski E., King V. M., Li X. (2002). Host circadian clock as a control point in tumor progression. *Journal of the National Cancer Institute*.

[6] Wright K. P., McHill A. W., Birks B. R., Griffin B. R., Rusterholz T., Chinoy E. D. (2013). Entrainment of the human circadian clock to the natural light-dark cycle. *Current Biology*.

[7] Sachdeva U. M., Thompson C. B. (2008). Diurnal rhythms of autophagy: implications for cell biology and human disease. *Autophagy*.

[8] Clairambault J., Gaubert S., Lepoutre T. (2011). Circadian rhythm and cell population growth. *Mathematical and Computer Modelling*.

[9] Xiang S., Coffelt S. B., Mao L., Yuan L., Cheng Q., Hill S. M. (2008). Period-2: a tumor suppressor gene in breast cancer. *Journal of Circadian Rhythms*.

[10] Preitner N., Damiola F., Zakany J., Duboule D., Albrecht U., Schibler U. (2002). The orphan nuclear receptor REV-ERB*α* controls circadian transcription within the positive limb of the mammalian circadian oscillator. *Cell*.

[11] Xiao L., Chang A. K., Zang M.-X. (2014). Induction of the CLOCK gene by E2-ER*α* signaling promotes the proliferation of breast cancer cells. *PLoS ONE*.

[12] Filipski E., Li X. M., Lévi F. (2006). Disruption of circadian coordination and malignant growth. *Cancer Causes and Control*.

[13] Sothern R. B., Levi F., Haus E., Halberg F., Hrushesky W. J. M. (1989). Control of a murine plasmacytoma with doxorubicin-cisplatin: dependence on circadian stage of treatment. *Journal of the National Cancer Institute*.

[14] Kelleher F. C., Rao A., Maguire A. (2014). Circadian molecular clocks and cancer. *Cancer Letters*.

[15] Matsuo T., Yamaguchi S., Mitsui S., Emi A., Shimoda F., Okamura H. (2003). Control mechanism of the circadian clock for timing of cell division in vivo. *Science*.

[16] Gewirtz D. A. (1999). A critical evaluation of the mechanisms of action proposed for the antitumor effects of the anthracycline antibiotics adriamycin and daunorunicin. *Biochemical Pharmacology*.

[17] Balsalobre A., Damiola F., Schibler U. (1998). A serum shock induces circadian gene expression in mammalian tissue culture cells. *Cell*.

[18] Yagita K., Tamanini F., Yasuda M., Hoeijmakers J. H. J., van der Horst G. T. J., Okamura H. (2002). Nucleocytoplasmic shuttling and mCRY-dependent inhibition of ubiquitylation of the mPER2 clock protein. *The EMBO Journal*.

[19] Chen S.-T., Choo K.-B., Hou M.-F., Yeh K.-T., Kuo S.-J., Chang J.-G. (2005). Deregulated expression of the *PER1*, *PER2* and *PER3* genes in breast cancers. *Carcinogenesis*.

[20] Langmesser S., Tallone T., Bordon A., Rusconi S., Albrecht U. (2008). Interaction of circadian clock proteins PER2 and CRY with BMAL1 and CLOCK. *BMC Molecular Biology*.

[21] Eide E. J., Woolf M. F., Kang H. (2005). Control of mammalian circadian rhythm by CKI*ε*-regulated proteasome-mediated PER2 degradation. *Molecular and Cellular Biology*.

[22] Yang X., Wood P. A., Ansell C., Hrushesky W. J. M. (2009). Circadian time-dependent tumor suppressor function of period genes. *Integrative Cancer Therapies*.

[23] Bjarnason G. A., Jordan R. C. K., Sothern R. B. (1999). Circadian variation in the expression of cell-cycle proteins in human oral epithelium. *American Journal of Pathology*.

[24] Granda T. G., Liu X.-H., Smaaland R. (2004). Circadian regulation of cell cycle and apoptosis proteins in mouse bone marrow and tumor. *The FASEB Journal*.

[25] Zeng Z.-L., Luo H.-Y., Yang J. (2014). Overexpression of the circadian clock gene bmal1 increases sensitivity to oxaliplatin in colorectal cancer. *Clinical Cancer Research*.

[26] Goto H., Tomono Y., Ajiro K. (1999). Identification of a novel phosphorylation site on histone H3 coupled with mitotic chromosome condensation. *The Journal of Biological Chemistry*.

[27] Tsai M.-Y., Wiese C., Cao K. (2003). A Ran signalling pathway mediated by the mitotic kinase Aurora A in spindle assembly. *Nature Cell Biology*.

[28] Crosio C., Fimia G. M., Loury R. (2002). Mitotic phosphorylation of histone H3: spatio-temporal regulation by mammalian Aurora kinases. *Molecular and Cellular Biology*.

[29] Hrushesky W. J. M. (1985). Circadian timing of cancer chemotherapy. *Science*.

[30] Wang C.-Y., Wen M.-S., Wang H.-W. (2008). Increased vascular senescence and impaired endothelial progenitor cell function mediated by mutation of circadian gene Per2. *Circulation*.

[31] Jackson J. G., Pereira-Smith O. M. (2006). Primary and compensatory roles for RB family members at cell cycle gene promoters that are deacetylated and downregulated in doxorubicin-induced senescence of breast cancer cells. *Molecular and Cellular Biology*.

